# Indents

**Published:** 1908-05-15

**Authors:** 


### INDENTS.

☞Brief Articles of Techinical or Scientific Value will receive notice and comment. Contributions invited.

**What Shall Be Our Attitude Toward a Child with Irregular Teeth and with Poor Parents?**

No one realizes the difficulty of this situation more than I, nor do I believe any set rule of conduct can be formulated. It must be decided in each individual case according to the conditions and the conscience of the practitioner. I can hardly have the courage or the conviction to ask of the profession to do for these children what I did for many years when I was accepting cases of orthodontia. I never yet turned away a patient from my office on account of fees where there was an earnest desire on the parents' part to have the teeth regulated, and I have in several instances carried through quite extensive cases of regulating without any fee at all. This I now believe to have been wrong—both in principle and in practice. It is demoralizing to any individual to accept something for nothing, and the man who fosters such a thing is guilty of an injustice to society. There are few parents today so very poor that they cannot pay something for such service, and the work will be more appreciated if it involves some expense. A perfectly frank understanding should accordingly be had with the parent to the effect that there must be a mutual sacrifice, and I am convinced that in the end every conscientious practitioner will be better pleased with himself if he carries through a case of that kind on the most approved principles, even though his fee is not large.

The great trouble with this entire question of compensation is that too many professional men fail to develop early in their career that perfect understanding and complete confidence between practitioner and patient which is really the keystone in the arch of professional harmony and goodwill. When a patient knows that a dentist is honest and capable and has in his makeup that essential element to all perfect success—the milk of human kindness—there is little difficulty concerning the regulation of fees, provided the patient really wishes the dentist's services. No professional man shall allow financial considerations to influence him to do an operation which may work a permanent or irreparable injury to the patient.

This rule carried out to the letter, and backed up by the spirit of loving kindness which should permeate all professional effort, will develop a delightful sentiment between practitioner and patient, and I am yet to be convinced that it will in the end leave the practitioner any poorer either in spirit or in purse.—C. N. Johnson, in *Review*.

**Diet, Health and Fletcherism.** Within recent years the question of the relationship between diet and health has given rise to renewed active discussion in medical and lay circles. At periodical intervals apparently new discoveries are made by some one who has made a study of the food requirements which furnishes the needed total calorific energy for the pursuance of the daily routine work. In recent lay literature "Fletcherism" is the latest phase of this much discussed question. A simple and terse description of what is meant by the term Fletcherism is given in one of the lay journals of recent date. It consists briefly in chewing all food taken into the mouth to a milky consistency, chewing it, tasting it, enjoying it, until it is involuntarily swallowed. All fluids that have flavor should be sipped. The suggestions contained in this epitaph are evident—it is merely a proof of the old adage: Eating too much fills the churchyard faster than eating too little. However, knowing things and practicing the same are different. There is probably no doubt that all of us fully agree to the above axioms, but to put them to practice is another matter. Nevertheless, it is good policy to put these simple truths forward under new names or in new shapes. They will always find some followers, because the thing seems to be a novelty and they will do some good for a time.

The results which Mr. Fletcher claims to have accomplished with his method of chewing his food are certainly remarkable. He is nearly 60 years of age and on two occasions in 1907 he has done hard work in emergencies which are significant as to his physical condition. Once he followed Major General Wood in the Philippines in climbing a volcanic mountain through a tropical jungle on an island for nine hours and at another time he waded through deep snow in the Himalayan mountains, some three miles one day and seven miles the next day in about as many hours. Professor Chittenden, of Yale University, has taken a deep interest in Mr. Fletcher's method of living, especially in regard of restricting the amount of daily food and in the tests made with the dynamometer on Mr. Fletcher and on eighteen Yale students. The former doubled the best record made by any trained athlete. Dr. Anderson of the Yale gymnasium says: "During the thirty-five years of my experience in physical training and teaching I have never tested a man who equaled this record. The latter tests, given in June, 1907, were more taxing than those given in 1903, but which Mr. Fletcher underwent with more apparent ease than he did four years ago. What seems to me a most remarkable feature of Mr. Fletcher's tests is that a man nearing 60 years of age should show progressive improvement of muscular quality merely as a result of dietic care and with no systematic physical training."

The results of Mr. Fletcher's demonstrations are very significant regarding the principle of properly chewing the food. The functionary factors for the proper performance of this principle—good sound teeth—are however, the most essential necessities in the pursuance of this newly (?)established fad.—*Dental Era.*

**Enthusiasm Should be Tempered with Judgment.** In the practice of dentistry it is well at all times to be on our guard, and as far as possible avoid using new methods and materials until their usefulness has been fully demonstrated. From time to time we have gone through periods of extreme changes, generally resulting in many grave mistakes which proved to be detrimental both to the practitioner and to the community. We believe we are in just such a period of change at the present time, and we might date this back to the beginning of the general practice of porcelain inlay work. An examination of this practice for the last ten years will certainly show great extremes and many mistakes wherein porcelain was adopted when gold should have kept its place. This is such an acknowledged fact now that it does not need discussion here, but the relief that has now come from the new process of casting gold fillings, brought out so prominently by Dr. Taggart less than a year ago, has come at an opportune time, as practitioners were casting about to find some relief or method to take the place of the failures of porcelain; and especially was this true in large restorations. But now what is following?

Great extremes and defective operations growing out of —first, carelessness and a lack of proper knowledge of the preparation of the cavity. Second, the misconceived idea that the operation is easy. When the practitioner adopts the idea or acts on the belief that the fitting of a filling of wax, which is to be replaced by gold when cast, is an easy part of the operation, he is bound to have what accuracy would call a failure. Many practitioners are using wax which is easily shaped and bent and making a restoration which is bound to prove a misfit when completed, if for no other reason than that the wax was not a proper fit when made. If one will fit wax into a cavity out of the mouth and examine carefully these wax restorations under a magnifying glass, it will be discovered that there are many gaps, here and there, away from the edges of the cavity, which, when completed in gold, and especially when cemented, will show defects and generally at points where accuracy and close fitting are absolutely essential. If this be true in making the wax fillings out of the mouth, how much more care should be exercised and observed when fitting them in extensive cavities in the teeth in the mouth. We contend that a wax suitable for this purpose should be stiff and unyielding; that will break at the sharp edges rather than bend away from the place.

It is a mistake also to insert cast fillings in moderate cavities which could be done quicker and more perfectly by the ordinary process of gold foil packed filling. This is true for two reasons: First, as we have observed that it can be done quicker and more accurately; and, second, it can be done in many cases without sacrificing so much tooth structure, which is necessary in the making of cast gold restorations in order to be able to remove the wax filling without disturbing it. The very best material and the very best instruments that can be made for casting are not too good and are essential for the perfect casting of these gold restorations in difficult cavities; and we contend, generally speaking, that these are the only cavities in which these methods should be adopted. This same proposition will also hold true in regard to the adaptation of artificial crowns to the roots of badly decayed teeth by the casting process, which in our opinion is one of the most valuable applications of the method, and is a great improvement over the old method of banding the root by cutting it down and forcing the band under the margin of the gums.

It is not our intention to dampen the enthusiasm of the profession over this new method, but to call attention to these precautions with a view of lessening mistakes and failures which are liable to bring a most useful method into disrepute. Use the casting process in all of its applications, but temper your enthusiasm with good, sound judgment.—J. N. Crouse, in *Digest*.

